# The association between mammography reports and women’s age using retrospective data form a medical system

**DOI:** 10.3389/fgwh.2025.1561669

**Published:** 2025-06-10

**Authors:** Wen-Mi Chang, Yi-Chun Chen, I-Cheng Lu, Jen-Lung Chen, Hung-Yi Chuang

**Affiliations:** ^1^ Outpatient Department, E-DA DaChang Hospital, I-Shou University, Kaohsiung, Taiwan; ^2^ Department of Health Management, I-Shou University, Kaohsiung, Taiwan; ^3^ Department of Occupational Medicine, E-DA Hospital and College of Medicine, I-Shou University, Kaohsiung, Taiwan; ^4^ Division of General Surgery, Department of Surgery, E-DA Hospital, I-Shou University, Kaohsiung, Taiwan; ^5^ Department of Public Health, Department of Environmental and Occupational Medicine, Kaohsiung Medical University Hospital, Kaohsiung Medical University, Kaohsiung, Taiwan

**Keywords:** mammography, BI-RADS, female, breast cancer, age, BMI, marital status

## Abstract

**Objective:**

We explored the association of age, obesity and marital status with Breast Imaging Reporting and Data System (BI-RADS) category distribution in women, using retrospective mammography data from a medical system, as only a few studies have investigated this association.

**Materials and methods:**

This retrospective study collected the first left-lateral and right-lateral mammography data of 4,165 and 4,213 women from 2,011 to 2020 in a medical system, respectively, to be analyzed. We examined the association of age, body mass index (BMI), and marital status with BI-RADS categories using the Chi-square test and multinomial logistic regression.

**Results:**

The proportion of BI-RADS category 0 decreased with age, but the proportion of BI-RADS categories 4 and 5 increased with age. Women aged >69 years had a higher proportion of BI-RADS categories 4 and 5 [adjusted odds ratio (aOR) = 1.34 for left-lateral mammography and aOR = 1.29 for right-lateral mammography data] than women aged 45–69 years. Overweight was associated with the rate of the proportion of BI-RADS categories 4 and 5 of the left-lateral breast (aOR = 1.26, *p* < 0.05) but not with the right-lateral breast.

**Conclusions:**

Age >69 years, being overweight, and being separated/divorced/widowed were the factors associated with BI-RADS categories 4 and 5 of women who underwent mammography.

## 1 Introduction

It is believed that early diagnosis of breast cancer can reduce the mortality rate by approximately 20%–40% (1, 2). Mammography is commonly used to detect breast cancer in its early stages (3, 4). Mammography data interpretation and classification are based on the Breast Imaging Reporting and Data System (BI-RADS) (5). According to the BI-RADS 5th version, BI-RADS category of 0 is recommended combining ultrasound or other examination to clarify the current status; categories of 1, 2, and 3 needing regular follow-up; and categories of 4 and 5 needing tissue diagnosis, that is, biopsy (5).

However, the sensitivity of mammography was associated with breast density (6, 7). Carney et al. analyzed seven population-based mammography registry data in the United States (6). They found that mammography sensitivity increased from 62.9% in women with high breast density to 87.0% in women with low breast density (6). A previous study found that more than a quarter of women with dense breasts were misdiagnosed during mammography because of the masking of lesions (6).

Women's breast density was found to be inversely associated with age (8, 9). It was reported that almost 75% of women aged 40–49 years had dense breasts, and the percentage decreased to 57% when women's age was over 50 years old. Heller et al. found a negative trend between breast density and age when women were younger than 56 years (9).

In addition, obesity is an identified risk factor for breast cancer (10–12) in postmenopausal women. A 13-year follow-up study of postmenopausal women in the US found that the risk of invasive breast cancer increased with increasing Body Mass Index (BMI) (10). A case-control study in Egypt reported that the risk of breast cancer was 2.28 times in overweight/obese postmenopausal women compared with underweight/normal-weight postmenopausal women (12).

Marital status was mentioned associated with the willing to undergo mammography and with breast cancer (13–15). Jolidon et al. found married women were more willing to undergo mammography compared with women without a partner or those living alone (13). Widowed women were associated with breast cancer (14) and had higher probability of being diagnosed with breast cancer at a later stage (15). Therefore, in the present study, we aimed to investigate the association of the BI-RADS category distribution with age, obesity and marital status among women in Taiwan using retrospective 10-year mammography data from a medical system to survey the BI-RADS category distribution.

## 2 Materials and methods

### 2.1 Sampled mammography data

The retrospective study was used in this study. The mammography data from 2011 to 2020 were collected from a medical system in southern Taiwan because the government provides the same mammography screening program during this period. Considering that each person had a different number of mammography data, only data of women's first mammogram in this medical system were included to be analyzed. The mammography data without the BI-RADS categorized were excluded in this study.

We also collected data on age, height, weight, and marital status from the participant's medical records at the time of the first mammogram in this medical system. The data of height and weight were measured and recorded and marital status was self-reported. The overall left and right lateral mammography data of 4,165 and 4,213 women, respectively, were included and analyzed.

### 2.2 Data analysis

According to the BI-RADS 5th version, different BI-RADS categories have different corresponding recommendation (5). Therefore, we categorized the results of mammography as BI-RADS “0,” “1,2,3,” and “4,5”. Body mass index (BMI) was calculated as weight (kg) divided by height squared (m^2^) and classified into underweight (BMI < 18.5), normal weight (18.5 ≤ BMI < 24), overweight/ obese (24 ≤ BMI), according to the recommendation of the Taiwan Health Promotion Administration (16). In this study, we categorized age into the following groups: <45 years, 45–69 years, and >69 years as the government provides a free biennial mammography screening program for women aged 45–69 years and for women aged 40–44 years whose relatives (grandmother, mother, or sister) have had breast cancer (17); marital status was categorized as married, unmarried, and separated/divorced/widowed.

We first examined the distribution of age, BMI, marital status, and BI-RADS of mammography data. Then, the association of BI-RADS categories distribution with age, BMI, and marital status was examined using a chi-square test. The odds ratios (ORs) and 95% confidence intervals (CIs) were calculated using univariate- and multivariate-multinomial logistic regression analysis with a main effect model to examine the association of BI-RADS categories with age, BMI classification, and marital status. In this multinomial logistic regression analysis, BIRADs “1,2,3,” age 45–69 years, normal BMI, and married were used as references. Left- and right-lateral mammography data were examined separately. We set the α value at 0.05, and data analyses were performed using SPSS software version 18 (IBM Corp., Armonk, NY, USA).

### 2.3 Ethical approval

This study was approved by the Research Ethics Committee of E-Da Hospital (EMRP-111-009). Informed consent was not required because retrospective data was used in this study.

## 3 Results

### 3.1 Age distribution, BMI, and marital status of study objects

About 75.8% of women between the ages of 45–69 underwent left-lateral mammography, over 49% were overweight/obese, 78.4% were married, 12.0% were classified into BI-RADS category 0, and 10.2% were classified as BI-RADS categories 4 and 5. About 75.7% of women aged 45–69 years underwent right-lateral mammography, overweight/obese were 49.2%, 77.9% were married, 11.7% were classified into BI-RADS category of 0, and 9.9% were classified of BI-RADS categories 4 and 5 (Table 1).

**Table 1:** Distribution of age, BMI, material status, and BI-RADS categories of women who underwent mammography.

Variables	Left-lateral mammography (*n* = 4,165)	Right-lateral mammography (*n* = 4,213)
	*n* (%)	*n* (%)
Age, years		
<45	320 (7.7)	322 (7.6)
45–69	3,155 (75.8)	3,189 (75.7)
>69	690 (16.6)	702 (16.7)
BMI classification		
Underweight	153 (3.7)	163 (3.9)
Normal	1,990 (47.8)	1,978 (46.9)
Overweight/obese	2,022 (49.3)	2,072 (49.2)
Marital status		
Married	3,267 (78.4)	3,284 (77.9)
Unmarried	338 (8.1)	342 (8.1)
Others^a^	560 (13.5)	587 (14.0)
BI-RADS category		
0	498 (12.0)	492 (11.7)
1,2,3	3,242 (77.8)	3,305 (78.4)
4,5	425 (10.2)	416 (9.9)

^a^ Including being widowed, separated and devoiced.

### 3.2 Description of the Bi-RADS categories associated with age, BMI, and marital status

The association of BI-RADS categories with age, BMI classification, and marital status in women who underwent mammography are presented in Table 2. The proportion of women with a BI-RADS category 0 decreased with increasing age in the bilateral mammography data; on the contrary, as the age increased, the proportion of those with BI-RADS categories 4 and 5 increased and showed statistically significant differences (*p* < 0.05). Marital status also showed a statistically significant association with the BI-RADS categories (*p* < 0.05). The proportion of those with BI-RADS categories 4 and 5 was the highest among women with other marital status (separated/divorced/widowed) than married and unmarried women. In bilateral mammography, data showed that the proportion of BI-RADS categories 4 and 5 of overweight/obese women were higher than underweight and normal-weight women. However, BMI was not associated with the BI-RADS categories.

**Table 2:** BI-RADS categories associated with age, BMI, and material status in women who underwent mammography.

Variables	Left-lateral BI-RADS (*n* = 4,165)			Right-lateral BI-RADS (*n* = 4,213)		
	0	1,2,3	4,5	0	1,2,3	4,5
	*n* (%)	*n* (%)	*n* (%)	*n* (%)	*n* (%)	*n* (%)
Age, years						
<45	53 (16.6)	249 (77.8)	18 (5.6)	54 (16.8)	245 (76.1)	23 (7.1)
45–69	365 (11.6)	2,476 (78.5)	314 (10.0)	365 (11.4)	2,519 (79.0)	305 (9.6)
>69	80 (11.6)	517 (74.9)	93 (13.5)	73 (10.4)	541 (77.1)	88 (12.5)
*p*-value*	<0.001			0.002		
BMI classification						
Underweight	15 (9.8)	124 (81.0)	14 (9.2)	17 (10.4)	129 (79.1)	17 (10.4)
Normal	239 (12.0)	1,572 (79.0)	179 (9.0)	236 (11.9)	1,563 (79.0)	179 (9.0)
Overweight/obese	244 (12.1)	1,546 (76.5)	232 (11.5)	239 (11.5)	1,613 (77.8)	220 (10.6)
*p*-value*	0.096			0.537		
Marital status						
Married	379 (11.6)	2,575 (78.8)	313 (9.6)	378 (11.5)	2,603 (79.3)	303 (9.2)
Unmarried	43 (12.7)	260 (76.9)	35 (10.4)	49 (14.3)	255 (74.6)	38 (11.1)
Others^a^	76 (13.6)	407 (72.7)	77 (13.8)	65 (11.1)	447 (76.1)	75 (12.8)
*p*-value*	0.016			0.032		

^a^ including being widowed, separated and devoiced.

* Chi-square test.

The results of univariate- and multivariate- multinomial logistic regression analysis of the association of age, BMI, and marital status with BI-RADS categories are shown in Table 3. Women aged <45 years who underwent mammography were associated with the BI-RADS category 0, the adjusted OR (aOR) was 1.47 [95% confidence interval (CI) = 1.06–2.04] in left-lateral mammography and aOR in right-lateral mammography was 1.46 (95% CI = 1.05–2.03).

**Table 3:** Multinomial logistic regression analysis of age, BMI, and marital status associated with BI-RADS categories.

Variables	BI-RADS			BI-RADS		
	0	1,2,3	4,5	0	1,2,3	4,5
	Odds ratios (95% CI)			Adjusted odds ratios (95% CI)^a^		
Left-lateral mammography (*n* = 4,165)						
Age, years						
<45	1.44 (1.05–1.98)	Ref	0.57 (0.35–0.93)	1.47 (1.06–2.04)	Ref	0.55 (0.33–0.92)
45–69	Ref	Ref	Ref	Ref	Ref	Ref
>69	1.05 (0.81–1.36)	Ref	1.42 (1.11–1.82)	1.01 (0.78–1.32)	Ref	1.34 (1.04–1.73)
BMI classification						
Underweight	0.80 (0.46–1.38)	Ref	0.99 (0.56–1.76)	0.78 (0.45–1.36)	Ref	0.99 (0.56–1.76)
Normal	Ref	Ref	Ref	Ref	Ref	Ref
Overweight/obese	1.04 (0.86–1.26)	Ref	1.32 (1.07–1.62)	1.05 (0.86–1.27)	Ref	1.26 (1.02–1.55)
Marital status						
Married	Ref	Ref	Ref	Ref	Ref	Ref
Unmarried	1.12 (0.80–1.58)	Ref	1.11 (0.76–1.61)	1.03 (0.72–1.46)	Ref	1.35 (0.92–1.98)
Others^b^	1.27 (0.97–1.66)	Ref	1.56 (1.19–2.04)	1.27 (0.97–1.66)	Ref	1.45 (1.10–1.91)
Right-lateral mammography (*n* = 4,213)						
Age, years						
<45	1.52 (1.11–2.08)	Ref	0.78 (0.50–1.21)	1.46 (1.05–2.03)	Ref	0.72 (0.46–1.14)
45–69	Ref	Ref	Ref	Ref	Ref	Ref
>69	0.93 (0.71–1.22)	Ref	1.34 (1.04–1.73)	0.94 (0.71–1.23)	Ref	1.29 (0.99–1.67)
BMI classification						
Underweight	0.87 (0.51–1.47)	Ref	1.15 (0.68–1.95)	0.85 (0.50–1.44)	Ref	1.15 (0.68–1.95)
Normal	Ref	Ref	Ref	Ref	Ref	Ref
Overweight/obese	0.98 (0.81–1.19)	Ref	1.19 (0.97–1.47)	1.01 (0.83–1.23)	Ref	1.15 (0.93–1.43)
Marital status						
Married	Ref	Ref	Ref	Ref	Ref	Ref
Unmarried	1.32 (0.96–1.83)	Ref	1.28 (0.89–1.84)	1.19 (0.85–1.67)	Ref	1.44 (0.99–2.10)
Others^b^	1.00 (0.76–1.33)	Ref	1.44 (1.10–1.89)	1.01 (0.76–1.35)	Ref	1.37 (1.04–1.80)

^a^ Model included age, BMI classification, and material status.

^b^ Including being widowed, separated and devoiced.

In the analysis of left-lateral mammography data, age >69 years (aOR = 1.34, 95% CI = 1.04–1.73), being overweight/obese (aOR = 1.26, 95% CI = 1.02–1.55), and being other marital status (aOR = 1.45, 95% CI = 1.10–1.91) were the factors associated with BI-RADS categories 4 and 5. However, only other marital status (aOR = 1.37, 95% CI = 1.04–1.80) were associated with BI-RADS categories 4 and 5 in right-lateral mammography data.

## 4 Discussion

In our study, the proportion of those with a BI-RADs category 0 was over 10%, and it decreased with age. The mammography of women classified into the BI-RADs category 0 was recommended to combine ultrasound or other examinations to clarify the current status of the breast. More than a quarter of women with dense breasts were misdiagnosed during mammography owing to the masking of lesions (7). The breast density decreased with increasing age (8, 9). Young women, particularly Chinese women, have dense breasts, which may lead to misclassifications in mammography (9). As people age, breast density decreases, and breast abnormalities become easier to detect through mammography. Carney et al. found the sensitivity of mammography from 68.6% in women aged 40–44 years increased to 83.3% in women aged 80–89 years (6).

Age was positively associated with the proportion of BI-RADs categories 4 and 5. This result was similar to a five-year community-based breast cancer screening results in Turkey (18). The mammography of women classified into the BI-RADs category 4 and 5 were advised to undergo tissue diagnosis, that is, biopsy. Compared with women aged 45 to 69 years, the ORs of breast biopsy among women aged >69 years were 1.42 and 1.34 for left- and right-lateral breasts, respectively. Noonpradej et al. found that positive predictive rate of breast cancer increased with age of women with BI-RADS category 4 (19).

Checka et al. found that more than 35% of women aged over 70 years still had dense breasts (8). Women aged 70–84 years had a higher risk (OR = 1.31) of diagnosis of breast cancer at a later stage than women in screening ages (50–69 years) in Swiss (15). Women older than 69 years, no longer eligible for free mammography programs provided by the government may experience delays in seeking medical services. Besides, according to the Nutrition and Health Survey in Taiwan from 2017 to 2020, the overweight and obesity rate was approximately 42.8% in women aged over 18 years and the rate increased with age in Taiwan (20). Obesity was mentioned as a risk factor for postmenopausal breast cancer (10–12). This implied that women aged >69 years may have a higher likelihood of receiving mammography results in BI-RADS categories 4 and 5.

In the current study, being overweight/obese was associated with BI-RADS categories 4 and 5 in the left-lateral mammography data analysis. In the right-lateral mammography data analyzed, the ORs of overweight/obese women showed little higher values than that of the normal-weight women, however, without statistical significance. The asymmetry of bilateral breast may cause this result. Abdou et al. (2022) found compared with right-lateral breast, several cell proliferation gene sets more enriched on the left-lateral breast (21). Women who underwent mammography and were classified as BI-RADS categories 4 and 5 were recommended further biopsy to have tissue diagnosis.

Obesity was reported to be associated with breast cancer (10–12, 22). A US follow-up study of postmenopausal women found that the risk of invasive breast cancer increased with increasing Body Mass Index (BMI) (10). A Singapore study found that the attributable risk proportion of breast cancer associated with BMI was 16.2%; women with a breast density <12% and maintaining a BMI <25 kg/m^2^ could decrease the breast cancer rate by 45.9% (22). Overweight and obese women who underwent mammography and were classified into BI-RADS categories 4 and 5 should follow the doctor's recommendation for further biopsy or combining other examinations to determine whether they have breast cancer and receive treatment as soon as possible.

Being separated/divorced/widowed was associated with the BI-RADS categories 4 and 5 in our study. Compared with married women, widows and separated or divorced women might have less financial and emotional support, which may result in them neglecting their physical health. In a previous study, married women were more willing to undergo mammography than women without a partner or those living alone (13). A Swiss population-based study showed that single/widowed/divorced women had a higher probability of being diagnosed with breast cancer at a later stage than married women (15). Duijts et al. found that the death of a spouse was associated with breast cancer. In addition, widows and separated or divorced women might experience more life stress than married women (14). Such stress may affect their immune system and, subsequently, breast health. Lawrence et al. (2023) found that life stress was associated with estrogen receptor-negative breast cancer (23).

To the best of our knowledge, few studies have investigated the factors associated with the BI-RADS categories in women through mammography data analysis. Our study provides valuable information that may help women aged >69 years, who are separated/divorced/widowed, or who are overweight and obese to be concerned about their breast health.

## 5 Conclusions

This study highlighted that women aged >69 years, and being overweight/ obese, being widowed/ separated/ divorced were the factors associated with the BI-RADS categories 4 and 5 (needing further tissue diagnosis, that is, biopsy) through mammography. Nowadays, with the life expectancy increasing, even women over 69 should be concerned about their breast health and have regular mammography for early detection and early treatment.

## 6 Limitations

This study had few limitations. Retrospective mammography data and medical records were used in this study; limited by the data, only a few confirmed variables could be analyzed. Otherwise, the mammography data were interpreted. Although hospital radiologists receive regular professional training, there is still a possibility of misclassification.

## Data Availability

The data analyzed in this study is subject to the following licenses/restrictions: We use de-identified mammography data in this paper; however, the data belongs to E-Da medical system and application is required. Requests to access these datasets should be directed to kimi@isu.edu.tw.

## References

[1] Lotter W Diab AR Haslam B Kim JG Grisot G Wu E Robust breast cancer detection in mammography and digital breast tomosynthesis using an annotation-efficient deep learning approach. Nat Med. (2021) 27:244–9. 10.1038/s41591-020-01174-9 33432172 PMC9426656

[2] Seely JM Alhassan T. Screening for breast cancer in 2018—what should we be doing today? Curr. Oncol. (2018) 25:115–24. 10.3747/co.25.3770 PMC6001765 29910654

[3] Chang CC Ho TC Lien CY Shen DHY Chuang KP Chan HP The effects of prior mammography screening on the performance of breast cancer detection in Taiwan. Healthcare. (2022) 10:1037. 10.3390/healthcare10061037 35742089 PMC9223050

[4] Kuo CS Chen GR Hung SH Liu YL Huang KC Cheng SY. Women with abnormal screening mammography lost to follow-up: an experience from Taiwan. Medicine (Baltimore). (2016) 95:e3889. 10.1097/MD.0000000000003889 27310983 PMC4998469

[5] Spak DA Plaxco JS Santiago L Dryden MJ Dogan BE. BI-RADS® fifth edition: a summary of changes. Diagn Interv Imaging. (2017) 98:179–90. 10.1016/j.diii.2017.01.001 28131457

[6] Carney PA Miglioretti DL Yankaskas BC Kerlikowske K Rosenberg R Rutter CM Individual and combined effects of age, breast density, and hormone replacement therapy use on the accuracy of screening mammography. Ann. Intern. Med. (2003) 138:168–75. 10.7326/0003-4819-138-3-200302040-00008 12558355

[7] Okello J Kisembo H Bugeza S Galukande M. Breast cancer detection using sonography in women with mammographically dense breasts. BMC Med Imaging. (2014) 14:41. 10.1186/s12880-014-0041-0 25547239 PMC4311471

[8] Checka CM Chun JE Schnabel FR Lee J Toth H. The relationship of mammographic density and age: implications for breast cancer screening. AJR Am J Roentgenol. (2012) 198:W292–5. 10.2214/AJR.10.6049 22358028

[9] Heller SL Hudson S Wilkinson LS. Breast density across a regional screening population: effects of age, ethnicity and deprivation. Br J Radiol. (2015) 88:20150242. 10.1259/bjr.20150242 26329467 PMC4743450

[10] Neuhouser ML Aragaki AK Prentice RL Manson JE Chlebowski R Carty CL Overweight, obesity, and postmenopausal invasive breast cancer risk: a secondary analysis of the women’s health initiative randomized clinical trials. JAMA Oncol. (2015) 1:611–21. 10.1001/jamaoncol.2015.1546 26182172 PMC5070941

[11] Britt KL Cuzick J Phillips K-A. Key steps for effective breast cancer prevention. Review Nat Rev Cancer. (2020) 20:417–36. 10.1038/s41568-020-0266-x 32528185

[12] Kamal RM Mostafa S Salem D ElHatw AM Mokhtar SM Wessam R Body mass index, breast density, and the risk of breast cancer development in relation to the menopausal status; results from a population-based screening program in a native African-arab country. Acta Radiol Open. (2022) 11:20584601221111704. 10.1177/20584601221111704 35795247 PMC9252007

[13] Jolidon V De Prez V Bracke P Bell A Burton-Jeangros C Cullati S. Revisiting the effects of organized mammography programs on inequalities in breast screening uptake: a multilevel analysis of nationwide data from 1997 to 2017. Front Public Health. (2022) 10:812776. 10.3389/fpubh.2022.812776 35198524 PMC8858931

[14] Duijts SF Zeegers MP Borne BV. The association between stressful life events and breast cancer risk: a meta-analysis. Int J Cancer. (2003) 107:1023–9. 10.1002/ijc.11504 14601065

[15] Feller A Schmidlin K Bordoni A Bouchardy C Bulliard JL Camey B Socioeconomic and demographic disparities in breast cancer stage at presentation and survival: a Swiss population-based study. Int J Cancer. (2017) 141:1529–39. 10.1002/ijc.30856 28657175

[16] Health Promotion Administration, Taiwan. The recommendation values of body mass index of children and adolescents. Taiwan: Health Promotion Administration (2023). Available at: https://www.hpa.gov.tw/Pages/Detail.aspx?nodeid=542&pid=9737 (Accessed September 06, 2023).

[17] Health Promotion Administration, Ministry of Health and Welfare. 2021 Health Promotion Administration Annual Report. Taiwan: Health Promotion Administration, Taiwan (2021). p. 93–6. Available at: https://www.hpa.gov.tw/Pages/Detail.aspx?nodeid=4519&pid=14657 (Accessed July 25, 2023).

[18] Çevik C. An examination of five-year community-based breast cancer screening results: a retrospective descriptive study. Acıbadem Univ Sağlık Bilim Derg. (2024) 15:181–5. 10.31067/acusaglik.1399163

[19] Noonpradej S Wangkulangkul P Woodtichartpreecha P Laohawiriyakamol S. Prediction for breast cancer in BI-RADS category 4 lesion categorized by age and breast composition of women in Songklanagarind hospital. Asian Pac J Cancer Prev. (2021) 22(2):531–6. 10.31557/APJCP.2021.22.2.531 33639670 PMC8190358

[20] Pan WH. Report of nutrition and health survey in Taiwan (2017-2020). (2021). Available online at: https://www.hpa.gov.tw/File/Attach/15562/File_18775.PDF (accessed on 08 07 2023).

[21] Abdou Y Gupta M Asaoka M Attwood K Mateusz O Gandhi S Left sided breast cancer is associated with aggressive biology and worse outcomes than right sided breast cancer. Sci Rep. (2022) 12:13377. 10.1038/s41598-022-16749-4 35927418 PMC9352772

[22] Ho PJ Lau HSH Ho WK Wong FY Yang Q Tan KW Incidence of breast cancer attributable to breast density, modifiable and non-modifiable breast cancer risk factors in Singapore. Sci Rep. (2020) 10:503. 10.1038/s41598-019-57341-7 31949192 PMC6965174

[23] Lawrence WR McDonald JA Williams F Shiels MS Freedman ND Lin Z Stressful life events, social support, and incident breast cancer by estrogen receptor status. Cancer Prev Res (Phila). (2023) 16:259–67. 10.1158/1940-6207.CAPR-22-0472 37067915 PMC10159918

